# Exploring Patterns of Alteration in Alzheimer's Disease Brain Networks: A Combined Structural and Functional Connectomics Analysis

**DOI:** 10.3389/fnins.2016.00380

**Published:** 2016-09-07

**Authors:** Fulvia Palesi, Gloria Castellazzi, Letizia Casiraghi, Elena Sinforiani, Paolo Vitali, Claudia A. M. Gandini Wheeler-Kingshott, Egidio D'Angelo

**Affiliations:** ^1^Department of Physics, University of PaviaPavia, Italy; ^2^Brain Connectivity Center, C. Mondino National Neurological InstitutePavia, Italy; ^3^Department of Electrical, Computer and Biomedical Engineering, University of PaviaPavia, Italy; ^4^Department of Brain and Behavioural Sciences, University of PaviaPavia, Italy; ^5^Neuroradiology Unit, C. Mondino National Neurological InstitutePavia, Italy; ^6^Brain MRI 3T Mondino Research Center, C. Mondino National Neurological InstitutePavia, Italy; ^7^NMR Research Unit, Queen Square MS Centre, Department of Neuroinflammation, UCL Institute of NeurologyLondon, UK

**Keywords:** AD, connectomics, disconnection syndrome, probabilistic tractography, resting state networks

## Abstract

Alzheimer's disease (AD) is a neurodegenerative disorder characterized by a severe derangement of cognitive functions, primarily memory, in elderly subjects. As far as the functional impairment is concerned, growing evidence supports the “disconnection syndrome” hypothesis. Recent investigations using fMRI have revealed a generalized alteration of resting state networks (RSNs) in patients affected by AD and mild cognitive impairment (MCI). However, it was unclear whether the changes in functional connectivity were accompanied by corresponding structural network changes. In this work, we have developed a novel structural/functional connectomic approach: resting state fMRI was used to identify the functional cortical network nodes and diffusion MRI to reconstruct the fiber tracts to give a weight to internodal subcortical connections. Then, local and global efficiency were determined for different networks, exploring specific alterations of integration and segregation patterns in AD and MCI patients compared to healthy controls (HC). In the default mode network (DMN), that was the most affected, axonal loss, and reduced axonal integrity appeared to compromise both local and global efficiency along posterior-anterior connections. In the basal ganglia network (BGN), disruption of white matter integrity implied that main alterations occurred in local microstructure. In the anterior insular network (AIN), neuronal loss probably subtended a compromised communication with the insular cortex. Cognitive performance, evaluated by neuropsychological examinations, revealed a dependency on integration and segregation of brain networks. These findings are indicative of the fact that cognitive deficits in AD could be associated not only with cortical alterations (revealed by fMRI) but also with subcortical alterations (revealed by diffusion MRI) that extend beyond the areas primarily damaged by neurodegeneration, toward the support of an emerging concept of AD as a “disconnection syndrome.” Since only AD but not MCI patients were characterized by a significant decrease in structural connectivity, integrated structural/functional connectomics could provide a useful tool for assessing disease progression from MCI to AD.

## Introduction

Alzheimer's disease (AD) is a neurodegenerative disorder characterized by a severe derangement of cognitive functions, typically in elderly subjects. The pathological hallmarks are the accumulation of β amyloid (Aβ1–42) plaques and tau tangles, mainly in the prefrontal and mesial-temporal lobes, respectively (Braak and Braak, 1995). Structural changes culminate in neuronal death and white matter degeneration, such as AD is characterized by progressive diffuse cortical atrophy primarily located in the mesial-temporal regions (Yao et al., 2010; Amlien and Fjell, 2014). Classically, pathological changes localized in discrete brain regions can support alterations of specific cognitive functions (Brier et al., 2014), while more complex neuropsychological deficits may be explained by the disconnection between brain regions with coordinated activity. The notion of a “disconnection syndrome” was originally introduced by Geschwind (1965) as referring to those pathologies where an event (e.g., a stroke) disconnects distinct brain regions and originates cognitive dysfunctions. Recent MRI studies have reported evidence supporting this disconnection hypothesis in AD patients, e.g., demonstrating changes in density of white matter associative fibers, meaning that the cognitive decline in AD may be caused by abnormalities in functional and anatomical interactions among different brain regions belonging to brain networks, rather than deficits localized to isolated brain areas (Bozzali et al., 2011; Brier et al., 2014). However, it is unclear whether the changes in functional connectivity are accompanied by corresponding structural connectivity changes.

Several neuroimaging studies using resting-state functional MRI (rs-fMRI) have revealed alterations in resting state networks (RSNs) of patients with AD and MCI. Most of these studies have focused on the default mode network (DMN) revealing reduced functional connectivity in the precuneus and posterior cingulate cortex (Binnewijzend et al., 2012; Toussaint et al., 2014; Zhong et al., 2014), while a few others have investigated the ensemble of RSNs demonstrating a diffuse impairment of functional connectivity going well beyond DMN (Agosta et al., 2013; Song et al., 2013; Castellazzi et al., 2014). These studies have revealed both reduced and increased functional connectivity in AD patients while a marked prevalence of functional connectivity increase has been demonstrated in MCI patients (Castellazzi et al., 2014). Alongside functional studies, several investigations have assessed the impairment of structural connections. Diffusion tensor imaging (DTI) and tractography have demonstrated decreased structural integrity through indices such as fractional anisotropy (FA), which was found to be reduced in hippocampal and posterior-anterior connections (Palesi et al., 2012; Wegrzyn et al., 2013), including the superior longitudinal and fronto-occipital fasciculi (Acosta-Cabronero and Nestor, 2014), supporting the idea that not just cortical areas but also subcortical connections running in the white matter are involved in cognitive decline of AD and MCI patients. Despite these findings, it is unclear whether the reported bi-directional changes in functional connectivity and white matter structural alterations of patients are correlated.

Recently, new approaches based on the graph theory (Rubinov and Sporns, 2010; Sporns, 2011) have been proposed to investigate topological changes in structural and functional networks in humans *in vivo*. Graph theory, a branch of mathematics increasingly applied to human sciences, provides metrics characterizing relevant properties of the networks such as *efficiency, integration*, and *segregation*. In order to define the topology of brain networks, it is essential to define their *nodes* and *edges*. The most intuitive way is to define a node as a particular cortical region of interest (ROI) while an edge as a connection between a pair of nodes. Edges, in turns, can be defined in several different ways, e.g., by using inter-region time series correlations from functional data or tractography streamlines (Bassett and Bullmore, 2006; Bullmore and Sporns, 2009; Sporns, 2011).

Some studies have used graph theoretical analysis to correlate the alterations of functional brain networks in AD patients with the pathophysiology of the disease (Supekar et al., 2008; Xiang et al., 2013), while others have focused on how structural (He et al., 2008) and white matter brain networks are impaired in these patients (Bai et al., 2012; Daianu et al., 2013). These efforts generally showed abnormal global topology in structural networks of AD patients but the results were substantially inconsistent, showing a different pattern of structural networks involvement in each study. Furthermore, only a few studies have investigated the relationship between functional and structural brain networks on the same cohort of subjects (Hagmann et al., 2008; Honey et al., 2009; Sun et al., 2014; Wang et al., 2015), generally by assessing the correlation between graph theoretical measurements of the functional and structural networks. To our knowledge, no studies were conducted on AD and MCI patients to address changes in the topological properties of structural networks that had been built using results from fMRI data analysis.

We have hypothesized that cognitive deficits in AD and MCI patients are associated to functional and structural alterations extending beyond the areas typically damaged by extensive neurodegeneration. Furthermore, since studies on healthy subjects (Horn et al., 2014; Van Oort et al., 2014) have revealed that functional connectivity reflects the underlying structural connectivity of specific circuits, we have speculated that structural/functional alterations in AD and MCI patients may be characterized by a more complex pattern than a simple one-to-one relationship. To test this hypothesis, we adopted a multi-parametric approach, i.e., rs-fMRI networks showing major changes in patients vs. controls were used to seed DTI tractography performed in the same subjects, thus allowing the reconstruction of a combined structural/functional connectomic. Indeed, for the first time on a cohort of AD and MCI patients, our exploratory study combined a commonly accepted tractography approach, which uses rs-fMRI results for seeding tractography, with graph theory for inferring topological proprieties of the brain networks under consideration. By doing so, we were able to assess the structural brain changes inside the same networks that were found to be functionally impaired in AD and MCI (Castellazzi et al., 2014) and to investigate the alterations of network connectivity in cross-sectional populations of subjects at different stages of the disease.

## Materials and methods

In a previous work (Castellazzi et al., 2014), 26 subjects with diagnosis of AD or MCI along with a matched group of 16 HC were studied and their RSN alterations were characterized using rs-fMRI data. In the same groups of subjects, DTI data were also acquired. In this study, the relationship between RSN alterations and the potential underlying structural changes is analyzed using the nodes of the most altered RSNs as seeds for tractography, thus allowing the reconstruction of a combined structural/functional connectomics.

### Experimental design

A total of 42 subjects was analyzed using OpenEpi online tool (Dean AG, Sullivan KM, Soe MM. OpenEpi: Open Source Epidemiologic Statistics for Public Health, Version 3.01, http://www.openepi.com/) to achieve sufficient statistical power (more than 80% tested on several network metrics) to differentiate AD patients from HC.

**Figure 1:**
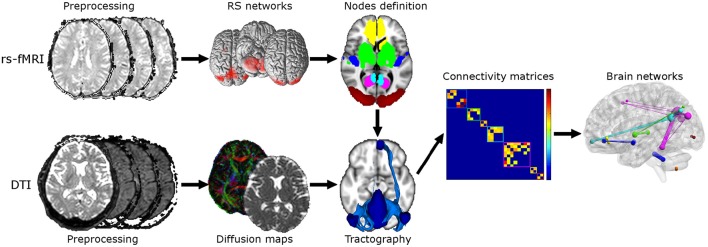
**Flow chart of the experimental design**. Top: Steps for the rs-fMRI analysis consisting of standard preprocessing, RSNs identification, and nodes definition. Bottom: Steps for the DTI analysis consisting of standard preprocessing, diffusion maps creation (e.g., FA, MD) and tractography between each pair of nodes. Twenty-nine ROIs were derived from RSNs analysis and used for tractography reconstruction. Middle right: steps for the graph theoretical analysis consisting in constructing connectivity matrices and calculation of graph theoretical measurements. Six different connectivity matrices were constructed and weighted by mean connectivity, mean FA, mean MD, mean aD, mean RD, and volume of tracts.

A *post-hoc* power analysis was performed on the parameter “global efficiency” weighted by MD to assess the number of subjects needed to detect a 0.05 effect at 80% power when exploring possible subtle changes between HC and MCI. A flow chart of the overall experimental design is shown in Figure 1. The top row of Figure 1 shows the steps of the rs-fMRI analysis consisting of standard preprocessing, RSNs identification, and nodes definition for tractography and graph theoretical analysis. The bottom row of Figure 1 shows the steps of the DTI analysis consisting of standard preprocessing, diffusion maps creation, and tractography between pairs of nodes within each identified network. The output of rs-fMRI and DTI analyses feeds into the steps of the graph theoretical analysis as shown in the middle row on the right.

### Participants

The same subjects included in Castellazzi et al. (2014)—for rs-fMRI analysis—were studied here for structural/functional connectomics: 26 patients were selected among those suffering from subjective or objective memory complaint, attending the memory clinic of the C. Mondino National Neurological Institute, Pavia, Italy. To create a reference metric for our findings, 16 HC (10 females, mean age 69 ± 5 years) were recruited on a volunteer base through a local recreational association (“Argento Vivo,” Bereguardo). All subjects, or their lawful caregiver, provided their written informed consent to the study.

Age above 80 years, significant medical, neurological (different from AD), or psychiatric disease as well as significant cerebrovascular disease, i.e., with a score ≥4 on the Hachinski scale (Hachinski et al., 1975; Binnewijzend et al., 2012), were considered part of the exclusion criteria for the study. Based on the scores obtained in the neuropsychological assessment (see section below), patients (26 subjects) were divided in two groups: 14 patients (10 females, mean age 70 ± 6 years) were classified as AD (NINCDS2-ARDA criteria; McKhann et al., 2011) and 12 patients (8 females, mean age 74 ± 6 years) as MCI (Petersen et al., 2001, 2009).

### Clinical and neuropsychological examination

All 42 subjects (14 AD, 12 MCI, and 16 HC) underwent a clinical and neuropsychological standardized battery of tests, which evaluated different cognitive domains (Spinnler and Dall'Ora, 1987; Spinnler and Tognoni, 1987). Global cognitive status was assessed with Mini-Mental State Examination (MMSE; Folstein et al., 1975). Memory was evaluated using logic memory (Novelli et al., 1986) and Rey–Osterrieth Complex Figure recall Test (Osterrieth, 1944; Caffarra et al., 2002). Attentive function was assessed by Trail Making Test A and B (Reitan, 1958). Language tests included semantic and phonemic fluency (Novelli et al., 1986; Randolph et al., 1993). Visuoconstructional ability was evaluated using Rey–Osterrieth Complex Figure copy Test (ROCF-copy). The neuropsychological results have been described in Castellazzi et al. (2014; see **Table 2**). For each test age- and education-corrected scores were calculated from the raw scores. Only corrected scores were used in the statistical analysis.

### MRI acquisition

All data were acquired using a 1.5T MR Philips Intera Gyroscan (Philips Healthcare, Koninklijke, The Netherlands) with an 8-channel head (SENSE) third-party coil. For each subject rs-fMRI, DTI, and T1 volumetric scans were acquired. A fast field echo-planar imaging sequence was used for rs-fMRI with repetition time (TR)/echo time (TE) = 3000/60 ms, field of view (FOV) = 250 mm, voxel size = 2.2 × 2.2 × 4 mm^3^, 26 slices, 100 repeated volumes. DTI data were acquired using a single-shot spin echo echo-planar imaging sequence with TR/TE = 11800/70 ms, FOV = 224 mm, number of averages = 3, 2.5 mm isotropic voxel, 60 axial slices and applying diffusion gradients along 15 non-collinear directions with *b*-value = 900 s/mm^2^. For anatomical reference a high-resolution 3DT1-weighted volume was collected using a fast field echo sequence with TR/TE = 8.6/4 ms, flip angle = 8°, FOV = 240 mm, slice thickness = 1.2 mm, in-plane resolution = 1.25 × 1.25 mm^2^, and 170 sagittal slices.

### DTI and rs-FMRI preprocessing

All MRI data were analyzed using SPM8 (Wellcome Department of Cognitive Neurology, http://www.fil.ion.ucl.ac.uk/), Matlab R2011b (The MathWorks, Natick, Mass, USA http://www.mathworks.com/), and FSL (FMRIB Software Library, http://fsl.fmrib.ox.ac.uk/fsl/fslwiki/).

DTI data were analyzed by performing eddy current correction and brain tissue extraction (Smith, 2006) on the non-diffusion weighted image with FSL. Diffusion tensor was calculated and FA, mean diffusivity (MD), axial diffusivity (aD), and radial diffusivity (rD) maps were created.

Rs-fMRI images were analyzed as previously described in Castellazzi et al. (2014). Briefly, for each subject independent component analysis was carried out using MELODIC (Beckmann et al., 2005) to identify RSNs, and a non-parametric permutation test (dual regression technique) was applied to create and compare group-specific maps for each independent RSN in MNI152 standard space (Montreal Neurological Institute, McGill, USA). Statistical maps were family-wise error corrected applying threshold-free cluster enhancement. A statistical threshold of *p* ≤ 0.05 was considered significant.

RSNs identified in Castellazzi et al. (2014) as relevant to distinguish between patients were transformed into each individual diffusion space using the following registration pipeline: for each subject the non-diffusion weighted image was realigned to the high-resolution 3DT1-weighted image using a full-affine transformation (12 degrees of freedom, FLIRT, FSL; Jenkinson et al., 2002); the 3DT1-weighted image was normalized to the MNI152 template by using a non-linear transformation (FNIRT, FSL); the MNI normalization procedure and the affine registration were inverted and the final combined transformation was applied to the RSNs found to be the most discriminant among groups when looking at functional connectivity alterations. For each subject the 3DT1-weighted volume was segmented into white matter, gray matter, and cerebrospinal fluid for calculating the total intracranial volume as the sum of these tissue types.

### RSNs nodes identification and tractography

RSNs found to be mostly discriminant among groups (Castellazzi et al., 2014) were used in this study for selecting nodes for structural analysis. These networks were: anterior insular (AIN), basal ganglia (BGN), cerebellum (CBLN), frontal cortex (FCN), lateral visual (LVN), and DMN. Since fMRI analysis revealed two distinct resting state components for the DMN, one with increased (DMNi) and one with reduced (DMNr) functional connectivity in patients, here the two components were considered separately. All clusters of continuous voxels identified in each RSN, both in the left and right hemisphere, were considered as network nodes.

For each subject probabilistic tractography (probtrackx, DTIFIT, FSL; Behrens et al., 2007) was used to connect each pair of nodes, for each RSN separately, by generating 5000 streamlines per voxel with a step of 0.5 mm. All tractography reconstructions were constrained introducing an intermediate target ROI to avoid spurious tracts and improve their anatomical fidelity. In detail, a NOT ROI was placed in the middle sagittal plane for tracts that do not cross between hemispheres while a target (AND) ROI was placed in the corpus callosum for tracts crossing between hemispheres. For each HC, all tracts were normalized in MNI152 space applying the previous combination of full-affine and non-linear transformations, and binarised (Ciccarelli et al., 2003). For each pair of nodes a mean and thresholded tract was created by selecting voxels belonging to that specific tract and common to at least 60% of HC.

### Brain networks construction

As described in the previous section, the selected RSNs were investigated separately in virtue of their intrinsic functional coherence and independence from the other investigated RSNs. For each subject and for each network, *nodes* were identified with the distinct clusters of the analogous RSN. Following this procedure a total of 29 nodes were identified in both hemispheres: four for BGN, five for DMNi, eight for DMNr, two for FCN, two for LVN, six for AIN (three cerebral and three cerebellar), and two for CBLN (all cerebellar). The 24 forebrain nodes were kept separated from the five cerebellar nodes in tractography and connectomics, since crossing fibers could not be resolved using the current approach.

Mean tracts previously created in MNI152 space were transformed into each individual subject diffusion space by inverting the normalization procedure to define *edges* of the brain networks.

For each subject and for each RSN, six N × N matrices (N represents the number of nodes and is specific for each RSN) were created using diffusion and structural proprieties as weights: mean connectivity (expressed as ratio between number of streamlines within a voxel and total number of generated streamlines), mean FA, mean MD, mean aD, mean rD, and normalized volume (expressed as ratio with respect to total intracranial volume) of each tract were used. In this way, each element of the matrix is directly associated to a specific tract's propriety.

### Brain networks analysis

The topology of brain networks was characterized using graph theoretical metrics calculated with Brain Connectivity Toolbox (Rubinov and Sporns, 2010). For completeness, formulas of the investigated theoretical metrics are reported in the **Appendix**.

Basic characterization was achieved calculating general properties of each node such as *nodal degree*, and *nodal strength*. The former gives information about the number of connections while the latter quantifies the strength of the connections between a node and its neighbors.

Other important brain networks features are their integration and segregation properties. Network integration represents the ability of a network to rapidly combine information from distributed brain regions. The most robust measure giving this kind of information is the *global efficiency*, which describes how well information is shared globally within the network.

Network segregation, which represents the ability for specialized processing to occur locally within densely interconnected regions, is usually expressed by the mean weighted clustering coefficient and the local efficiency of each node. The *weighted clustering coefficient* is calculated for each node and represents the percentage of the node's neighbors having other connections; the average value over all nodes indicates the extent of local interconnectivity in a network (Watts and Strogatz, 1998). Moreover, as well as global efficiency, the *local efficiency* represents the ability of sharing information but only with the direct neighbors of a specific node.

### Statistical analysis

A Shapiro-Wilk test was used to assess normality of the distribution of all brain network metrics, i.e., strength, global efficiency, clustering coefficient and local efficiency. For those normally distributed, a standard two-tailed one-way ANOVA test (*p* = 0.05) with Bonferroni correction for multiple comparisons was used to investigate differences among the groups. For metrics not normally distributed, a two-tailed Kruskall-Wallis test was used to compare measurements among the three groups while a Mann-Whitney *U*-test was used to investigate differences between two groups. Power calculations showed that in future studies the sample size should be increased to 37 subjects (21 HC and 16 MCI) in order to detect an effect size of 5% between these two groups in the global efficiency parameter, weighted with MD.

The relationship between cognitive performance and network metrics was evaluated using the following pipeline: Pearson correlations were performed between neuropsychological tests and network properties; the variables that emerged from this analysis were used in a multiple regression analysis. Here, all subjects were put together in one unique group. The significant neuropsychological tests were used as dependent variables while global and local efficiency, and mean strength were used as independent variables to assess which percentage of cognitive dysfunction they predict. For each dependent variable, two regression analyses were performed entering as independent variables: 1) each network metric individually; 2) global and local efficiency together to assess the global contribution of integration and segregation to cognitive dysfunction. These analyses were repeated for all different weights. The explained variance (*R*^2^) and its relative significance were calculated for each analysis.

## Results

Combined structural/functional connectomics (Figure 1) was carried out on the three groups of subjects, comprising HC, AD and MCI patients, that were previously described by Castellazzi et al. (2014). Reportedly, (i) age, gender distribution, and education did not reveal any significant differences among groups, (ii) significant differences (*p* < 0.05) between-groups were found for all the neuropsychological tests except for ROCF-copy, (iii) AD patients performed worst in all neuropsychological tests on cognitive functions, while MCI patients were worse than HC only in a limited set of tests, such as MMSE and Trail Making Test A (see Table 2 in Castellazzi et al. (2014) for details).

### Structural/functional brain networks analysis

**Figure 2:**
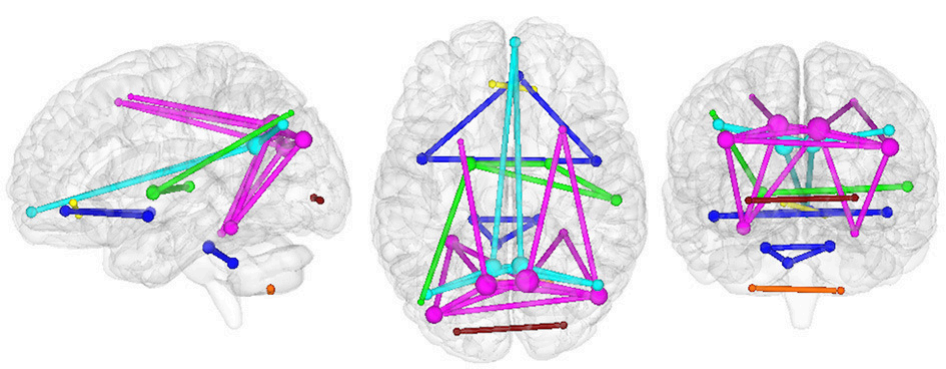
**Organization of investigated networks in HC in the three radiological planes**. Each color represents a different RSN: AIN (blue), BGN (green), CBLN (orange), DMNi (light blue), DMNr (fuchsia), FCN (yellow), and LVN (red). The size of each node is proportional to its nodal degree, while the thickness of each edge is set to one.

The present connectomic analysis explored seven structural/functional networks (Figure 2) comprising DMNi, DMNr, BGN, AIN, CBLN, FCN, and LVN.

**Figure 3:**
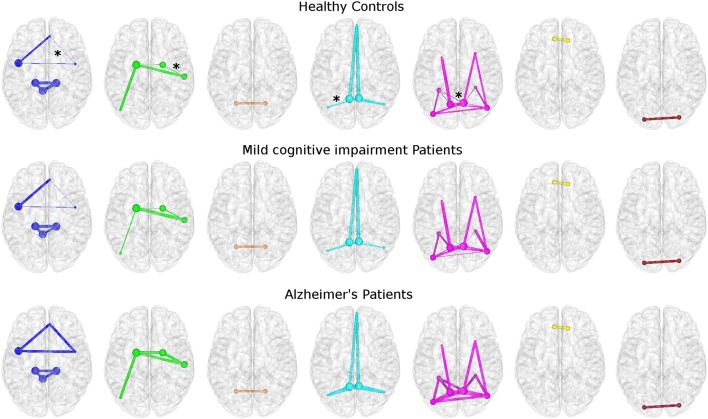
**Topological networks differences among groups**. Each row represents a different group: HC (top), MCI (middle), and AD (bottom). Stars identify edges with significant different strengths between HC and AD patients. All networks were built using mean aD as weight. From left: AIN, BGN, CBLN, DMNi, DMNr, FCN, and LVN. All networks are represented using the same color coding of Figure 2. The size of each node is proportional to its nodal degree. For HC and MCI patients the thickness of each edge corresponds to the normalized strength with respect to AD patients' one.

Topological features of brain networks were significantly different in AD patients with respect to HC. Smaller non-significant differences were found in MCI patients, either with respect to HC or AD patients (Figure 3). When comparing different properties of the edges of the networks between groups, mean connectivity and mean FA values along the tracts showed no difference in any network metrics. However, significant differences were found using other diffusion indices, e.g., MD and aD, to weight the edges as shown in Figure 3. Here, the thickness of the edges connecting the nodes in HC and in MCI patients is defined as the strength normalized to the same metric of AD patients.

In AD patients, when considering each RSN as an independent brain network, we observed the following:

- Alterations of network integration and segregation for multi-node networks, i.e., DMNi, DMNr, BGN, and AIN.
- No differences for networks consisting of two nodes, i.e., CBLN, FCN, and LVN.
- Significantly increased values of all network metrics when using MD and aD as weights in DMNi, DMNr, and BGN.
- Significantly increased values of all network metrics when using RD as weight in DMNi, DMNr.
- Significantly decreased clustering coefficient and local efficiency when using volume as weight in DMNi, DMNr, and AIN.

**Figure 4:**
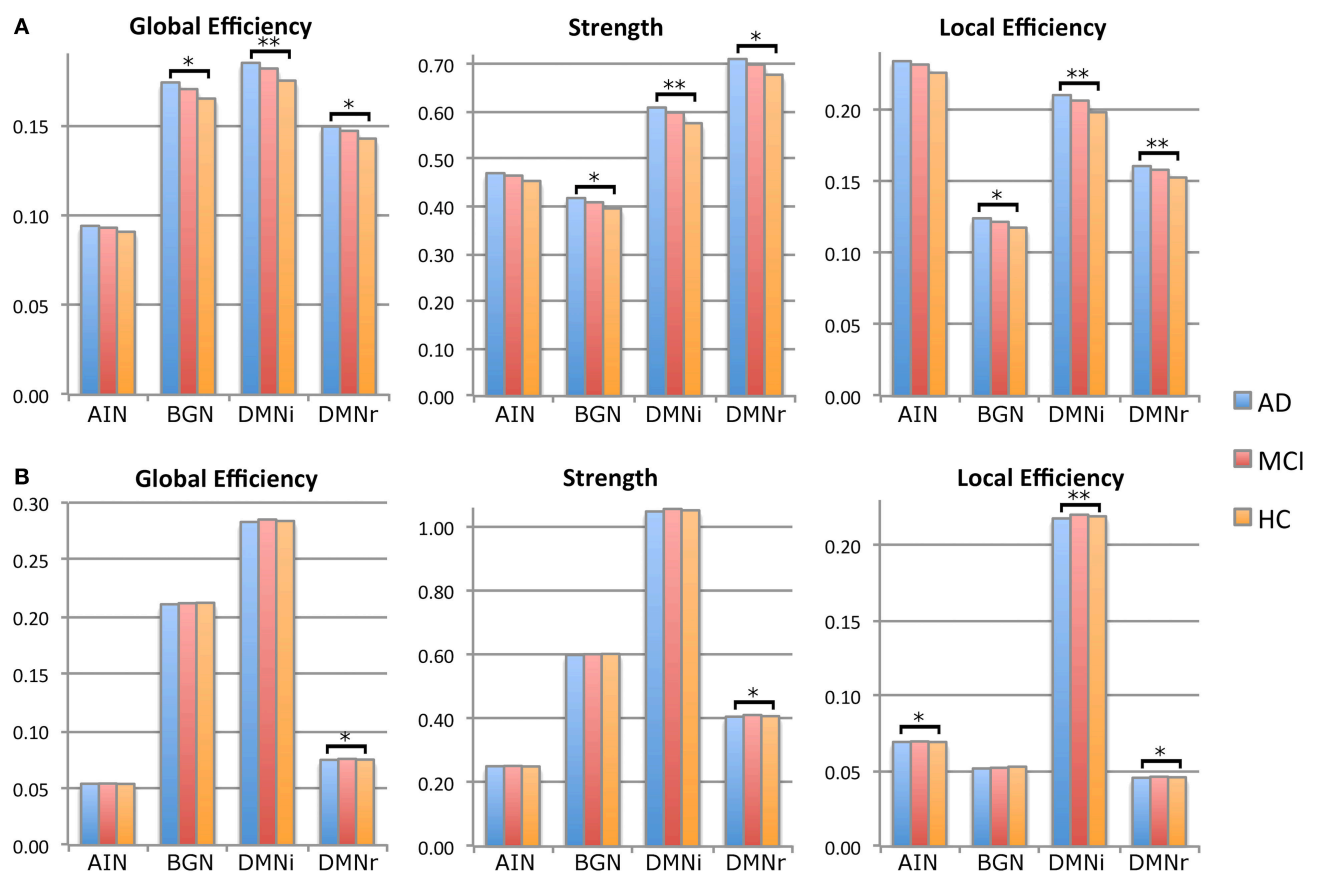
**Histograms of principal network measurements in the three groups of subjects: HC (orange), MCI (red), and AD (blue)**. Significance is reported at ^*^p < 0.05 and at ^**^p < 0.01. **(A)** Histograms calculated using mean aD as weight. DMNi shows the greatest significant difference with MCI and HC, followed by DMNr and BGN. AIN does not show any significant alteration. **(B)** Histograms calculated using mean volume as weight. DMNr shows significant alterations for all network parameters, while DMNi and AIN show significant alterations for local efficiency. BGN does not show any significant difference.

For the multi-node networks, only graph metrics (and relative *p*-value) that were significantly different between AD patients and HC are reported in Table 1. Detailed results including not significant differences between patients with MCI and HC are reported in supplementary data (Supplementary Table 1). All results were also graphically shown: Figure 4 shows results regarding metrics weighted by aD and volume while all other results are graphically reported in Supplementary Figure 1.

**Table 1:** **Graph theoretical measurements**.

			**Clustering coeff**		**Eglob**		**Strength**		**Eloc**	
			**Mean (*SD*)**	***p*-value**	**Mean (*SD*)**	***p*-value**	**Mean (*SD*)**	***p*-value**	**Mean (*SD*)**	***p*-value**
DMNi	MD	HC	0.157 (0.010)	**0.007**	0.162 (0.010)	**0.009**	0.535 (0.033)	**0.009**	0.186 (0.012)	**0.008**
		AD	0.169 (0.011)		0.173 (0.011)		0.573 (0.035)		0.200 (0.013)	
	aD	HC	0.166 (0.008)	**0.005**	0.175 (0.008)	**0.007**	0.575 (0.027)	**0.007**	0.198 (0.010)	**0.006**
		AD	0.176 (0.009)		0.185 (0.009)		0.609 (0.030)		0.210 (0.011)	
	rD	HC	0.141 (0.011)	**0.010**	0.144 (0.011)	0.013	0.476 (0.036)	0.012	0.167 (0.013)	0.011
		AD	0.154 (0.011)		0.155 (0.011)		0.515 (0.038)		0.181 (0.013)	
	Volume	HC	0.022 (0.001)	0.005^*^	0.029 (0.001)	n.s.	0.108 (0.004)	n.s.	0.023 (0.001)	0.005^*^
		AD	0.020 (0.001)		0.028 (0.001)		0.105 (0.004)		0.022 (0.001)	
DMNr	MD	HC	0.123 (0.007)	0.017	0.134 (0.007)	0.022	0.637 (0.036)	0.022	0.145 (0.008)	0.018
		AD	0.130 (0.008)		0.142 (0.008)		0.675 (0.040)		0.154 (0.009)	
	aD	HC	0.128 (0.006)	**0.010**	0.143 (0.006)	0.013	0.678 (0.028)	0.011	0.153 (0.007)	**0.010**
		AD	0.135 (0.007)		0.149 (0.007)		0.711 (0.034)		0.160 (0.008)	
	rD	HC	0.112 (0.008)	0.026	0.121 (0.008)	0.034	0.573 (0.040)	0.035	0.131 (0.009)	0.028
		AD	0.119 (0.009)		0.129 (0.009)		0.611 (0.044)		0.140 (0.010)	
	Volume	HC	0.004 (0.000)	0.023	0.008 (0.000)	0.045	0.043 (0.002)	0.042	0.005 (0.000)	0.025
		AD	0.004 (0.000)		0.007 (0.000)		0.041 (0.002)		0.005 (0.000)	
BGN	MD	HC	0.107 (0.006)	0.031	0.149 (0.009)	0.033	0.360 (0.022)	0.028	0.107 (0.006)	0.031
		AD	0.113 (0.007)		0.158 (0.009)		0.381 (0.022)		0.113 (0.007)	
	aD	HC	0.118 (0.005)	0.017	0.165 (0.008)	0.012	0.396 (0.018)	0.012	0.118 (0.005)	0.017
		AD	0.124 (0.007)		0.174 (0.009)		0.418 (0.022)		0.124 (0.007)	
AIN	Volume	HC	0.007 (0.000)	0.019	0.006 (0.000)	n.s.	0.026 (0.002)	n.s.	0.007 (0.000)	0.019
		AD	0.007 (0.000)		0.005 (0.000)		0.025 (0.001)		0.007 (0.000)	

Values are expressed as mean (SD). Statistical analysis: p < 0.05 was considered significant. Post-hoc pairwise comparisons were performed using Bonferroni or Mann-Whitney^*^. Bold p-values identify significances at p < 0.01..

### Structural/functional relationships

To explore possible pathophysiological mechanisms behind AD progression, the relationships between functional and structural alterations for several RSNs were investigated in patients with AD and MCI. Increases and decreases in both functional and structural alterations were compared.

Castellazzi et al. (2014) reported both increased and decreased functional connectivity in patients with AD and MCI for several RSNs. In particular, DMNi and AIN showed increased functional connectivity, DMNr showed decreased functional connectivity while BGN showed both areas of increased and decreased functional connectivity.

In the present study, for each investigated RSN, analysis of structural networks showed that graph metrics decreased in patients when FA and volumes were used as weights, while the same metrics increased in patients when MD, aD, and rD were used as weights. This trend is shown in Table 1 and in Supplementary Table 1.

### Relation between cognitive parameters and network metrics

**Figure 5:**
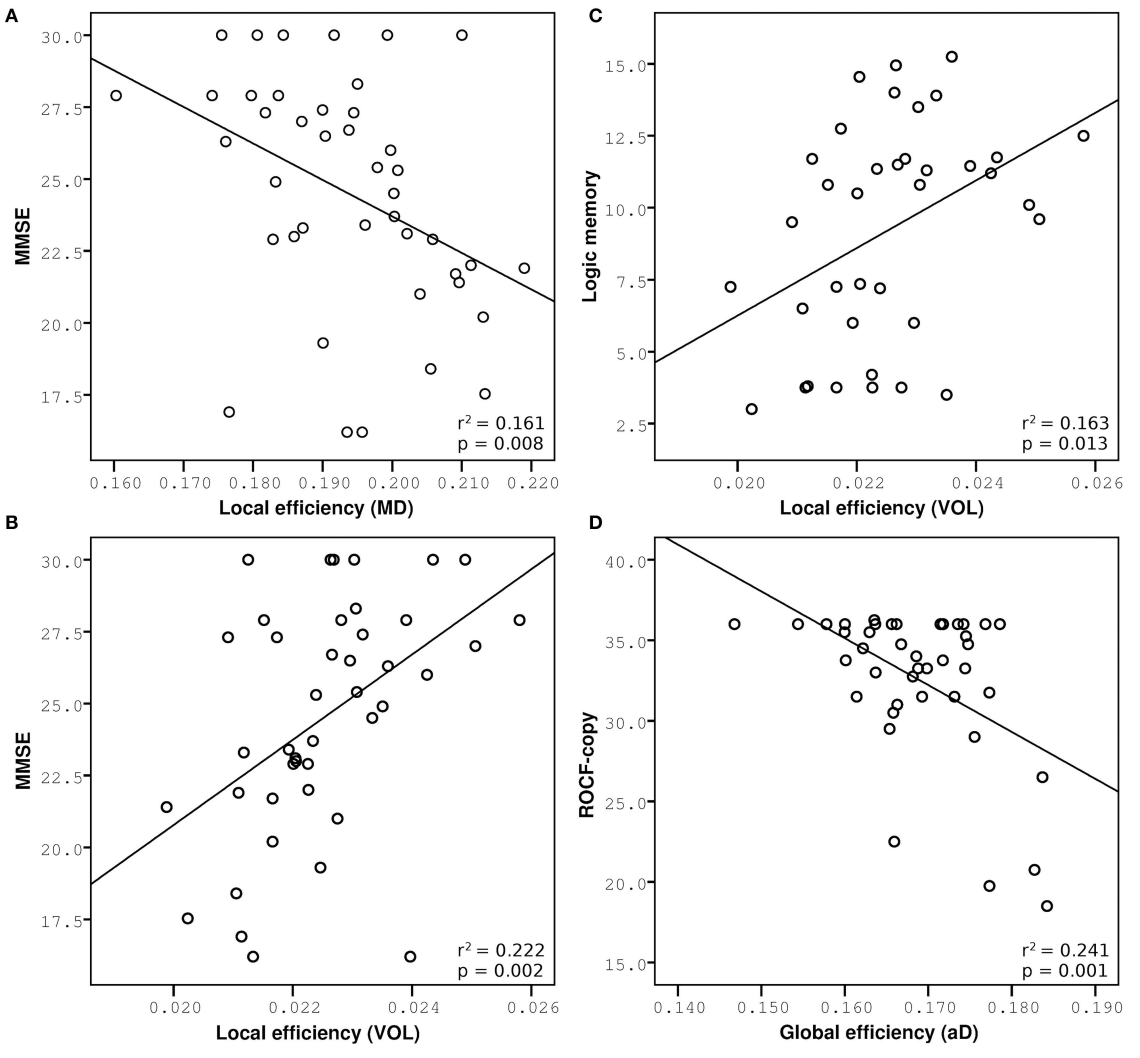
**Multiple regression analysis between network measurements and cognitive performance**. Increased network metrics indicate lower efficiency, expect for measures weighted by volume. Correlations of MMSE are reported on the left: **(A)** Negative correlation with local efficiency weighted by MD in DMNi, **(B)** positive correlation with local efficiency weighted by volume in DMNi. **(C)** Positive correlation between logic memory and local efficiency weighted by volume in DMNi. **(D)** Negative correlation between ROCF-copy and global efficiency weighted by aD in BGN.

To determine the relationship between brain alterations revealed by structural/functional connectomic analysis and the cognitive state of patients, the most relevant metrics were correlated with cognitive tests reported by Castellazzi et al. (2014). Pearson correlations verified that smaller network metrics were related with worse cognitive performance when tract volume and FA were used as weights, while increased metrics were related to worse cognitive performance when MD, aD, and rD were used as weights for each RSN. Three neuropsychological tests, i.e., MMSE, logic memory, and ROCF-copy, were selected as representative. Network metrics, i.e., global and local efficiency, explained partially the variance of these neuropsychological tests. All significant multiple regression analyses are reported in Table 2. Furthermore, to visually explain the most significant relations, major results of the multiple regression analysis are shown in Figure 5.

**Table 2:** **Regression analysis with graph theoretical metrics as predictors for performance in cognitive tests**.

		**Eglob**		**Strength**		**Eloc**		**Eglob + Eloc**	
		***R*^2^ (%)**	***p*-value**	***R*^2^ (%)**	***p*-value**	***R*^2^ (%)**	***p*-value**	***R*^2^ (%)**	***p*-value**
**MMSE**									
DMNi	MD	15.4	0.010	15.6	0.010	16.1	0.008	17.6	0.023
	aD	14.2	0.014	14.6	0.013	15.4	0.010	18.1	0.021
	rD	15.3	0.010	15.7	0.010	16.1	0.008	17.9	0.021
	Volume	ns	ns	ns	ns	22.2	0.002	24.6	0.004
DMNr	MD	13	0.019	13.8	0.015	13.6	0.016	ns	ns
	aD	13.9	0.015	14.9	0.012	15.0	0.011	15.7	0.036
	rD	12.3	0.023	12.8	0.020	12.6	0.021	ns	ns
	Volume	10.2	0.040	11.7	0.026	16.2	0.008	17	0.027
BGN	MD	14.6	0.013	14.9	0.011	14.8	0.012	15.0	0.042
	aD	18.1	0.005	17.5	0.006	14.6	0.013	20.0	0.013
	rD	11.7	0.026	12.6	0.021	14.2	0.014	14.4	0.048
AIN	Volume	ns	ns	ns	ns	9.2	0.05	ns	ns
**MEMORY PROSE**									
DMNi	MD	10.7	0.048	10.8	0.047	10.7	0.048	ns	ns
	rD	10.7	0.048	10.9	0.046	10.8	0.047	ns	ns
	Volume	ns	ns	ns	ns	16.3	0.013	16.9	0.043
DMNr	Volume	ns	ns	ns	ns	11.4	0.041	ns	ns
**ROCF-COPY**									
DMNi	FA	13.2	0.020	13.4	0.018	13.7	0.017	ns	ns
	MD	13.4	0.018	13.4	0.019	13.6	0.018	ns	ns
	aD	13.5	0.020	13.4	0.019	13.5	0.018	ns	ns
	rD	13.2	0.018	12.9	0.021	13.3	0.019	ns	ns
DMNr	FA	ns	ns	ns	ns	ns	ns	16.3	0.034
	MD	ns	ns	10.2	0.042	9.9	0.046	ns	ns
	aD	13.6	0.018	12.8	0.021	9.9	0.045	26.8	0.003
	rD	ns	ns	ns	ns	9.7	0.047	18.7	0.020
BGN	MD	23.1	0.001	21.9	0.002	17.9	0.006	25.5	0.004
	aD	24.1	0.001	23.2	0.001	20	0.003	25.9	0.003
	rD	21.1	0.002	19.9	0.003	15.3	0.011	23.4	0.006
AIN	FA	14.4	0.014	14.4	0.014	14.1	0.016	15.0	0.045

*R^2^-values are expressed as percentage*.

#### Prediction of global cognitive performance (MMSE)

Parameters weighted by MD, aD, rD explained partially the variance in MMSE for BGN, DMNi, and DMNr. Parameters weighted by volume explained a percentage of the variance in MMSE for DMNi and DMNr. For AIN, only local efficiency weighted by volume was significant. For each weight and for each RSN other than AIN, local, and global efficiencies together explained 2% more of the variance in MMSE rather than using network parameter individually.

#### Prediction of memory performance (logic memory)

Parameters weighted by MD or rD explained partially the variance in logic memory for DMNi, while local efficiency weighted by volume explained an amount of the variance in logic memory both for DMNi and DMNr. Local and global efficiencies together gave significant results only for DMNi when weighted by volume.

#### Prediction of visuoconstructional performance (ROCF-Copy)

Parameter weighted by MD, aD, or rD explained a percentage of the variance in ROCF-copy for BGN, DMNi, and DMNr. All parameters weighted by FA explained an amount of the variance in ROCF-copy for DMNi and AIN. For each weight and for each RSN other than DMNi, local, and global efficiencies together increased explanatory power.

## Discussion

This exploratory paper indicates that combined structural/functional connectomics could potentially be more informative on the nature of AD and MCI brain alterations than functional or structural connectomics alone. First, the connectivity reduction in AD patients goes beyond the areas primarily involved in neurodegeneration, affects in particular graph metrics of *integration* and *segregation* and correlates with cognitive impairment, thereby suggesting that further investigations with appropriate sample sizes should explore these metrics to confirm (or refute) the “disconnection syndrome” hypothesis of AD. Secondly, MCI patients do not show significant changes in structural connectivity, suggesting that their functional alterations affect large-scale brain loops before structural changes become evident. The study indeed confirms that the DMN is a crucial network in AD, but also shows that while a number of rs-fMRI networks were altered in MCI and AD, with specific patterns of increased and decreased functional connectivity, their structural substrate was altered mainly in AD, with structural connectivity of MCI subject being “not” significantly different to that of HCs. Thirdly, the pattern of structural alteration characterizes individual brain networks differently. These observations challenge the classical interpretation of AD based on the hypothesis that cognitive deficits are related to alterations in specific brain regions and imply that the pathogenesis of AD may well involve a hierarchy of changes, which could happen in parallel or in series, affecting possibly independently functional and structural properties of large-scale brain networks.

### Network-dependent alterations patterns

This study was carried out using a multi-parametric connectomic approach, based on the combination of spatially independent RSNs (resulting from the independent component analysis of rs-fMRI data: Castellazzi et al., 2014), tractography, and graph theoretical analysis to weight the edges of the networks and analyze their properties. The indices of overall diffusivity, such as MD, and of main diffusion components, such as aD, proved to be the most indicative when used for calculating graph theoretical measurements while tract volume was important when searching for local alterations. The large-scale network alterations revealed in this way affected the brain of AD patients extending to areas such as the white matter underlying parietal cortex and basal ganglia and provided evidence for white matter involvement and for disconnection among distinct brain regions in the pathology (Bai et al., 2012; Daianu et al., 2013). Different networks, such as DMN, BGN, and AIN, showed specific patterns of alterations for integration and segregation suggesting different levels of microstructure and macrostructure impairment. The MD, aD, and rD increase observed in neurodegenerative conditions (Parra et al., 2015) caused a corresponding change in network metrics, e.g., global and local efficiency. By using these indices as edge weights it turns out that the functional increases observed previously may reflect an unsuccessful attempt to compensate the pathological state rather than an effective compensation process for improving network performance. Overall, MD and aD increases proved especially informative about the pathological degree of alterations at network level in AD, possibly implying the presence of extended microstructural tissue degeneration.

The DMN was the RSN most affected by the pathology and showed alterations in integration, segregation and connections strength when considering both volumetric and diffusion properties as weights for network edges. In particular, higher network strength in patients was related to increased diffusivity, which suggests the presence of white matter degeneration at cellular and molecular level. Global efficiency is associated with long-range connections properties; therefore an increase of global efficiency may reflect a disrupted global integration of the structural networks in AD patients and points toward the presence of disconnected brain regions. Segregation, which tells how specialized information is shared between close regions, was also altered indicating that both local and global information sharing may be compromised along the posterior-anterior connections in patients. Moreover, network edges weighted by volume showed decreased values implying the presence of macrostructural degeneration, mainly involving the posterior-anterior connections. These findings, if confirmed by larger studies, could prove the strong involvement, both microstructural and macrostructural, of temporal-prefrontal connections in AD supporting the hypothesis that the pathology is related to altered connectivity among distinct brain areas.

Similarly to DMN, BGN showed alterations in integration, segregation and connections strength, although only diffusion proprieties provided significant results in patients with respect to HC. This suggests that the pathological processes involving connections between basal ganglia regions are mainly altering local microstructure rather than macrostructure.

Furthermore, volume was also the only useful weight detecting segregation alterations in AIN, which showed decrease clustering coefficient and local efficiency. This suggests that local sharing of information among brain regions involved in AIN is compromised because these structures are atrophic in AD patients while microstructural changes are not significant. This finding is in line with the concepts that AD affects primarily gray matter regions whereas white matter impairment is a secondary pathological effect.

### Structural/functional relationship

Our findings reveal that AD affects structural properties of the DMN, BGN, and AIN, which include brain regions known to be among the first to be involved in AD. Indeed, the principal hubs of these networks, i.e., the nodes with highest nodal degree, were located in the precuneus bilaterally and in the superior temporal lobe. In agreement with results from Castellazzi et al. (2014), alterations of these regions were indicated as fundamental in AD; nevertheless, our structural findings suggest that there may be a hierarchy of micro and macrostructural damages that underlies their functional alterations.

Castellazzi et al. (2014), by using the same cohort of subjects of the present study, reported both increase and decrease functional connectivity in AD patients suggesting the coexistence of two competing pathophysiological mechanisms, i.e., compensation and degeneration. However, it was also argued that increased functional connectivity might be the result of frequencies locking rather than of compensatory mechanisms. The present study, using diffusion MRI, confirms on microstructural basis that the edges of the main functionally impaired networks are characterized only by decreased FA and increased diffusivities indices supporting the presence of generalized structural degeneration (Acosta-Cabronero et al., 2012; Bosch et al., 2012). This result, together with the functional connectivity increases corresponding to a generalized structural degeneration, is against the hypothesis of efficient functional compensatory processes in these networks of AD patients. It is worth noting that the DMN, which is the most functionally and structurally affected network, could be affected by multiple neurodegenerative mechanisms, such as neuronal loss and axonal integrity disruption. The functional impairment of BGN and AIN could be explained by a single pathophysiological mechanism, i.e., disruption of white matter integrity in BGN and neuronal loss in AIN.

Network metrics of MCI patients showed only minor (not significant) structural alterations. Thus, the remarkable functional connectivity increases detected in their RSNs may indicate the presence of effective functional compensatory mechanisms engaging the cognitive reserve to compensate disease progression.

### Relationship between cognitive performances and network measurements

The observation that different patterns of alterations specifically affected different brain networks deserves further commenting. Structural connectivity within the DMN correlated with reduced performance in MMSE, which tests global cognitive decline. Similar correlations were previously observed between functional connectivity alterations of DMN and MMSE in the same patients (Castellazzi et al., 2014). This picture is in agreement with the function classically attributed to the DMN, which is related to global resting activity rather than to specific cognitive functions (Raichle, 2015). Interestingly, local efficiency weighted by MD and by volume had the highest explanatory power suggesting that global cognitive decline depends on several mechanisms and atrophy of the posterior-anterior connections could be a potential imaging biomarker of the AD pathological stage. It is worth noting that the explanatory power of local efficiency is around 20% meaning that changes in variance of MMSE can be only partially explained by changes of local efficiency. Indeed, MMSE scores corresponding to 30, i.e., the maximum value, are associated with a broad range of local efficiency values showing a “ceiling effect” in the correlation between these measures (see Figure 5A). This could depend on the presence of MCI subjects with a high cognitive reserve that is not captured by the MMSE score and may indeed confound data. This is typical of clinical and neuropsychological scores that are often unable to represent fine progression of a specific physical or cognitive decline. Nevertheless, this “ceiling effect” observed in Figures 5A,B may be a confound for the statistical power of the correlation that it is, however, highly significant.

Specific cognitive domains were further investigated performing regression analyses on logic memory and ROCF-copy tests. Damage in the memory domain, evaluated by logic memory, and its variance were not explained by network metrics, while they correlated with functional alterations in AIN (Castellazzi et al., 2014). Only the DMN revealed correlations with logic memory, according with the important role of the DMN in memory functions. Actually the best predictor of logic memory was identified in the volume-weighted local efficiency of a DMN component (the so called DMNi), further supporting the importance of volume-weighted analysis for the identification of local alterations in cognitive processing.

Visuoconstructional ability and praxis, evaluated through the ROCF-copy test, were well explained by microstructural degeneration. Indeed, network parameters weighted by diffusion indices explained about 15% of variance in the ROCF-copy test for all the investigated RSNs. It must be noted that a great number of subjects, including MCI and few AD patients, obtained the highest score in the ROCF-copy test (see Figure 5D), i.e., 36, suggesting that the ROCF-copy test may not be the ideal test for distinguishing different classes of subjects. It is known that the neuroanatomical network underlying praxis involves frontal and parietal cortices, basal ganglia, and their white matter connections (Leiguarda and Marsden, 2000; Gross and Grossman, 2008). Interestingly, our findings show that BGN was the network that better explained variance in the ROCF-copy test. This result could suggest that the role of basal ganglia in determining visuoperceptual and visuospatial deficits is very important and could be further investigated in AD patients.

### Methodological considerations

Some limitations should be considered for the present investigation. First of all, our DTI sequence was run in a clinical setting and acquired diffusion-weighted signals along 15 non-collinear directions. Advanced diffusion models, needed for resolving complex architectures, such as Q-ball (Tuch, 2004), diffusion spectrum imaging (Wedeen et al., 2008) or constrained spherical deconvolution (Tournier et al., 2007) require diffusion-weighted images with an angular resolution three times greater than that used here. Therefore, only tractography based on the diffusion tensor model could be used in the present study. This represents a limitation because it has been largely demonstrated that DT-based tractography is good for reconstructing large fiber bundles that do not cross other tracts, while fails to reconstruct fan-shaped tracts extending to the lateral areas of the cortex (Farquharson et al., 2013). By reconstructing tracts with well-defined seed and target ROIs (as opposed to a whole-brain connectomic approach), we were able to limit the downside of a sub-optimal acquisition protocol. On the other hand, diffusion tensor tractography combined with graph theory has already proven to be valid for characterizing brain topology and assessing alterations in brain networks in AD and MCI patients (Lo et al., 2010; Bai et al., 2012; Daianu et al., 2013; Sun et al., 2014). To limit possible errors in estimating properties of the reconstructed tracts, such as volume, possibly enhanced by the inherent limitations of diffusion tensor tractography, we built average tracts from HCs only, where individual tracts were binarised, thresholded, and only those voxels belonging to more than 60% of HCs, namely the medial part of the tracts, considered. In this contest, probabilistic tractography based on the diffusion tensor model was the best choice available for this study and limited us to study individual rs-fMRI network connectivity rather than more complex circuits or whole brain connectomics. It is worth noting that our fMRI analysis was performed using ICA for identifying RSNs at group level. This means that local smoothing was used to obtain average results for all subjects together in MNI-152 space. Therefore the different size and morphology of fMRI and DTI voxels doesn't affect results of tractography. Furthermore, networks edges were identified starting from mean tracts from controls: actually, original tracts reconstructed for each subject were not used, since patients' tracts may be biased due to the presence of pathological conditions, such as small punctual lesions, brain atrophy, degeneration of axonal bundles or demyelination process. Original tracts were visually compared with the analogous mean inverted tracts highlighting the problem that the location of the two analogous tracts was comparable but all patients' tracts had a larger extent. With this approach, though, all reconstructed tracts were anatomically plausible and consistent between subjects.

Future studies using acquisition schemes with higher angular resolution for the diffusion-weighted directions may help explaining several aspects that could not be tackled here. Indeed several studies have shown that the anatomical confidence of tractography, both in healthy and in clinical cases, is strongly improved by using advanced diffusion algorithms, such as the constrained spherical method discussed in (Farquharson et al., 2013). Clinically, it would be useful to consider the whole-brain connectomics based on advanced tractography and interactions among multiple RSNs and evaluate to what extent the small-world connectivity is altered in patients. In this contest it would be interesting to consider advanced approaches such as the *track-weighted functional connectivity* (TW-FC) method (Calamante et al., 2013). Similar to the approach used in this study, TW-FC aims at providing functional-structural description of the brain by combining rs-fMRI and tractography data. This method provides maps that describe the white matter connections associated with a given RSN, and their intensity in a given voxel reflects the functional connectivity associated with the underlying structural connectivity. This approach could be useful in quantitative voxel-wise comparison for assessing alterations in neurological disorders, such as AD and MCI. In particular, it could be useful for investigating complex circuits, including the cerebro-cerebellar loops (Castellazzi et al., 2014; Palesi et al., 2015), which showed functional changes in MCI and AD patients and were not possible to investigate using the current method. Further, improvements could be obtained by adopting alternative brain parcellation methods: some atlases based on functional activations have been proposed (Yeo et al., 2011; Gordon et al., 2016) and could be used instead of population-based results.

Another limitation of this study was the relatively small sample size that might have inflated the effect size as well as having limited the statistical power to detect alterations of some of the investigated brain network metrics in AD and MCI groups. It is important to underline that the same cohort of subjects was successfully used in a previous rs-fMRI study (Castellazzi et al., 2014) for studying functional connectivity changes in both patients groups. Also in the present investigation several network metrics showed significant differences between AD and HC (after correction for multiple comparisons), providing interesting, though exploratory, findings that are useful for characterizing connectivity alterations in these patients. Besides these considerations, further studies are needed to validate and to assess the reproducibility of our results with a large number of subjects.

As noted above the structural network differences found in this study between MCI and HC were not statistically significant. Since network properties alterations have been detected in this class of prodromic patients (Bai et al., 2012), our results could reflect the fact that our cohort of MCI patients is heterogeneous and contains both patients converting to AD in the clinical follow-up (converter) and patients with stable MCI or even reverting to normal cognition. The follow-up of these patients may inform on whether some structural changes actually occur in one subcategory (e.g., the converters), thus providing insights about disease progression. Ad-hoc sample size calculations based on this exploratory study indicate that with a few extra subjects one should be able to detect the presence of early structural alterations.

## Conclusions

This combined connectomic approach using structural and functional data obtained from the same group of patients provides a framework for the analysis of pathophysiological mechanisms of AD, presenting exploratory findings that, once confirmed with larger studies, may indeed support the “disconnection syndrome” hypothesis. This is based on these key elements worth reporting: this approach allowed us to indicate that structural alterations potentially affect large-scale brain networks in AD but not MCI patients, and that specific microstructural and macrostructural alterations affect these networks differently. Longitudinal studies of larger cohorts could inform on whether combined structural/functional connectomics is able to provide early insights about the conversion from MCI to AD. Furthermore this novel approach has the potential to be translated for the investigation of the correlation between functional and structural network changes in other brain diseases.

## Author contributions

FP, CGWK, and ED conceptualized the study. FP designed and performed the analyses with support from GC and LC. ES acquired all neuropsychological data helping for data interpretation. CGWK and ED provided support and guidance with data interpretation with clinical contribution of PV and LC. FP, CGWK, and ED wrote the manuscript, with comments from all other authors.

## Funding

This work was supported by C. Mondino National Neurological Institute of Pavia (5 per mille 2013) and University of Pavia to FP and GC. This work was supported by grants of European Union (Human Brain Project; HBP-604102) and of the Italian Ministry of Health (RF-INM-2008-114341 and RF-2009-1475845) to ED and by “Ricerca Corrente 2015” to ED and CGWK.

### Conflict of interest statement

The authors declare that the research was conducted in the absence of any commercial or financial relationships that could be construed as a potential conflict of interest.

## Appendix

### Brain networks analysis

For completeness, here are reported formulas for brain network analysis. Suppose that we are dealing with a network with *N* nodes along with a corresponding list of undirected edges connecting these nodes. Mathematically, this is described by an N × N binary adjacency matrix *A* whose *ij*_th_ element (*a*_*ij*_) is = 1 if nodes *i* and *j* are connected.

The *nodal degree* of node *i* is given by

$$\begin{array}{l} k_{i}=\sum_{j\in N}a_{ij}. \end{array}$$

When the adjacency matrix is not binarised and each element is proportional to a specific weight (*w*_*ij*_), e.g., mean FA, the *nodal strength* of node *i* is given by

$$\begin{array}{l} S_{i}=\underset{j\in N}{\sum }w_{ij} \end{array}$$

and quantifies the strength of the connections between a node and its neighbors.

Networks integration and segregation are usually described by global and local efficiency, and the clustering coefficient. For their definition, it is necessary to introduce the shortest path length matrix whose elements (*Lij*) represent the shortest lengths of the connection between nodes *i* and *j*. The *global efficiency* is defined as the arithmetic mean of the inverses of the shortest path lengths between a node and all other nodes of the network:

$$\begin{array}{l} Eglob=\frac{1}{N}\sum_{i\in N}\frac{\sum_{j\in N,j\neq i}{\left(L_{ij}\right)}^{-1}}{N-1}. \end{array}$$

The *weighted clustering coefficient* of node *i* is given by

$$\begin{array}{l} C_{i}^{w}=2\sum_{j,h\in N}\frac{{\left(w_{ij}w_{ih}w_{jh}\right)}^{1/3}}{k_{i}\left(k_{i}-1\right)}. \end{array}$$

Finally, the *local efficiency* of node *i* is given by

$$\begin{array}{l} {Eloc}_{i}=\sum_{j,h\in N,j\neq i}\frac{{\left(w_{ij}w_{ih}{\left[L_{jh}\left(N_{i}\right)\right]}^{-1}\right)}^{1/3}}{k_{i}\left(k_{i}-1\right)}. \end{array}$$

## Abbreviations

aD: axial diffusivity
AIN: anterior insular network
BGN: basal ganglia network
CBLN: cerebellar network
DMN: default mode network
DMNi: increased default mode network
DMNr: reduced default mode network
DTI: diffusion tensor imaging
FA: fractional anisotropy
FCN: frontal cortex network
LVN: lateral visual network
MD: mean diffusivity
MMSE: Mini-Mental State Examination
RD: radial diffusivity
rs-fMRI: resting state functional MRI
RSN: resting state network.
