# Supplementary material for: Exploring Patterns of Alteration in Alzheimer's Disease Brain Networks: A Combined Structural and Functional Connectomics Analysis
Source: Front Neurosci. 2016 Sep 7;10:380. doi: 10.3389/fnins.2016.00380 (PMC5013043; doi:10.3389/fnins.2016.00380)
Supplement: Supplementary file 1 [file Table1.DOCX]

**Supplementary Table 1. Graph theoretical measurements**

|  |  | **Clustering coefficient** | | | **Global Efficiency** | | | **Strength** | | | **Local Efficiency** | | |
| --- | --- | --- | --- | --- | --- | --- | --- | --- | --- | --- | --- | --- | --- |
|  |  | HC | MCI | AD | HC | MCI | AD | HC | MCI | AD | HC | MCI | AD |
|  |  | Mean (sd) | Mean (sd) | Mean (sd) | Mean (sd) | Mean (sd) | Mean (sd) | Mean (sd) | Mean (sd) | Mean (sd) | Mean (sd) | Mean (sd) | Mean (sd) |
| **DMNi** | ***FA*** | 0.283 (0.013) | 0.281 (0.009) | 0.277 (0.010) | 0.323 (0.016) | 0.323 (0.010) | 0.318 (0.012) | 1.052 (0.051) | 1.049 (0.032) | 1.035 (0.039) | 0.344 (0.016) | 0.342 (0.011) | 0.337 (0.012) |
|  | *p-value* |  | 1.000 | 0.582 |  | 1.000 | 0.885 |  | 1.000 | 0.837 |  | 1.000 | 0.617 |
|  | ***MD*** | 0.157 (0.010) | 0.165 (0.009) | 0.169 (0.011) | 0.162 (0.010) | 0.169 (0.009) | 0.173 (0.011) | 0.535 (0.033) | 0.559 (0.030) | 0.573 (0.035) | 0.186 (0.012) | 0.195 (0.010) | 0.200 (0.013) |
|  | *p-value* |  | 0.152 | ***0.007*** |  | 0.175 | ***0.009*** |  | 0.171 | ***0.009*** |  | 0.157 | ***0.008*** |
|  | ***aD*** | 0.166 (0.008) | 0.173 (0.007) | 0.176 (0.009) | 0.175 (0.008) | 0.182 (0.007) | 0.185 (0.009) | 0.575 (0.027) | 0.598 (0.024) | 0.609 (0.030) | 0.198 (0.010) | 0.206 (0.009) | 0.210 (0.011) |
|  | *p-value* |  | 0.106 | ***0.005*** |  | 0.119 | ***0.007*** |  | 0.116 | ***0.007*** |  | 0.108 | ***0.006*** |
|  | ***rD*** | 0.141 (0.011) | 0.149 (0.010) | 0.154 (0.011) | 0.144 (0.011) | 0.151 (0.010) | 0.155 (0.011) | 0.476 (0.036) | 0.500 (0.032) | 0.515 (0.038) | 0.167 (0.013) | 0.176 (0.011) | 0.181 (0.013) |
|  | *p-value* |  | 0.201 | ***0.010*** |  | 0.234 | ***0.013*** |  | 0.229 | ***0.012*** |  | 0.210 | ***0.011*** |
|  | ***Volume*** | 0.022 (0.001) | 0.021 (0.001) | 0.020 (0.001) | 0.029 (0.001) | 0.029 (0.001) | 0.028 (0.001) | 0.108 (0.004) | 0.107 (0.004) | 0.105 (0.004) | 0.023 (0.001) | 0.023 (0.001) | 0.022 (0.001) |
|  | *p-value* |  | 0.114* | ***0.005**** |  | 1.000 | 0.085 |  | 1.000 | 0.141 |  | 0.114* | ***0.005**** |
| **DMNr** | ***FA*** | 0.223 (0.011) | 0.221 (0.006) | 0.220 (0.009) | 0.267 (0.013) | 0.265 (0.008) | 0.266 (0.012) | 1.254 (0.059) | 1.242 (0.036) | 1.242 (0.053) | 0.271 (0.013) | 0.269 (0.007) | 0.268 (0.011) |
|  | *p-value* |  | 1.000 | 1.000 |  | 1.000 | 1.000 |  | 1.000 | 1.000 |  | 1.000 | 1.000 |
|  | ***MD*** | 0.123 (0.007) | 0.128 (0.006) | 0.130 (0.008) | 0.134 (0.007) | 0.140 (0.006) | 0.142 (0.008) | 0.637 (0.036) | 0.663 (0.031) | 0.675 (0.040) | 0.145 (0.008) | 0.151 (0.007) | 0.154 (0.009) |
|  | *p-value* |  | 0.192 | ***0.017*** |  | 0.211 | ***0.022*** |  | 0.218 | ***0.022*** |  | 0.201 | ***0.018*** |
|  | ***aD*** | 0.128 (0.006) | 0.133 (0.005) | 0.135 (0.007) | 0.143 (0.006) | 0.147 (0.005) | 0.149 (0.007) | 0.678 (0.028) | 0.699 (0.026) | 0.711 (0.034) | 0.153 (0.007) | 0.158 (0.006) | 0.160 (0.008) |
|  | *p-value* |  | 0.146 | ***0.010*** |  | 0.210 | ***0.013*** |  | 0.191 | ***0.011*** |  | 0.150 | ***0.010*** |
|  | ***rD*** | 0.112 (0.008) | 0.117 (0.006) | 0.119 (0.009) | 0.121 (0.008) | 0.127 (0.007) | 0.129 (0.009) | 0.573 (0.040) | 0.600 (0.033) | 0.611 (0.044) | 0.131 (0.009) | 0.138 (0.008) | 0.140 (0.010) |
|  | *p-value* |  | 0.236 | ***0.026*** |  | 0.232 | ***0.034*** |  | 0.252 | ***0.035*** |  | 0.250 | ***0.028*** |
|  | ***Volume*** | 0.004 (0.000) | 0.004 (0.000) | 0.004 (0.000) | 0.008 (0.000) | 0.008 (0.000) | 0.007 (0.000) | 0.043 (0.002) | 0.042 (0.002) | 0.041 (0.002) | 0.005 (0.000) | 0.005 (0.000) | 0.005 (0.000) |
|  | *p-value* |  | 1.000 | ***0.023*** |  | 1.000 | ***0.045*** |  | 1.000 | ***0.042*** |  | 1.000 | ***0.025*** |
| **AIN** | ***FA*** | 0.376 (0.014) | 0.373 (0.016) | 0.370 (0.011) | 0.152 (0.006) | 0.151 (0.006) | 0.149 (0.004) | 0.758 (0.029) | 0.754 (0.031) | 0.747 (0.022) | 0.376 (0.014) | 0.373 (0.016) | 0.370 (0.011) |
|  | *p-value* |  | 1.000 | 0.672 |  | 1.000 | 0.744 |  | 1.000 | 0.744 |  | 1.000 | 0.672 |
|  | ***MD*** | 0.213 (0.014) | 0.219 (0.015) | 0.222 (0.010) | 0.086 (0.006) | 0.088 (0.006) | 0.089 (0.004) | 0.428 (0.029) | 0.440 (0.030) | 0.447 (0.020) | 0.213 (0.014) | 0.219 (0.015) | 0.222 (0.010) |
|  | *p-value* |  | 0.686 | 0.166 |  | 0.708 | 0.172 |  | 0.708 | 0.172 |  | 0.686 | 0.166 |
|  | ***aD*** | 0.226 (0.012) | 0.231 (0.012) | 0.234 (0.009) | 0.091 (0.005) | 0.093 (0.005) | 0.094 (0.004) | 0.454 (0.023) | 0.465 (0.025) | 0.470 (0.018) | 0.226 (0.012) | 0.231 (0.012) | 0.234 (0.009) |
|  | *p-value* |  | 0.527 | 0.145 |  | 0.561 | 0.147 |  | 0.560 | 0.147 |  | 0.527 | 0.145 |
|  | ***rD*** | 0.192 (0.016) | 0.198 (0.016) | 0.202 (0.010) | 0.077 (0.006) | 0.079 (0.006) | 0.081 (0.004) | 0.385 (0.031) | 0.397 (0.032) | 0.405 (0.021) | 0.192 (0.016) | 0.198 (0.016) | 0.202 (0.010) |
|  | *p-value* |  | 0.817 | 0.190 |  | 0.819 | 0.196 |  | 0.818 | 0.196 |  | 0.817 | 0.190 |
|  | ***Volume*** | 0.007 (0.000) | 0.007 (0.000) | 0.007 (0.000) | 0.006 (0.000) | 0.005 (0.000) | 0.005 (0.000) | 0.026 (0.002) | 0.026 (0.001) | 0.025 (0.001) | 0.007 (0.000) | 0.007 (0.000) | 0.007 (0.000) |
|  | *p-value* |  | 0.494 | ***0.019*** |  | 0.397 | 0.066 |  | 0.481 | 0.068 |  | 0.494 | ***0.019*** |
| **BGN** | ***FA*** | 0.224 (0.010) | 0.223 (0.006) | 0.224 (0.006) | 0.330 (0.017) | 0.333 (0.006) | 0.333 (0.009) | 0.783 (0.039) | 0.788 (0.015) | 0.789 (0.022) | 0.224 (0.010) | 0.223 (0.006) | 0.224 (0.006) |
|  | *p-value* |  | 1.000 | 1.000 |  | 1.000 | 1.000 |  | 1.000 | 1.000 |  | 1.000 | 1.000 |
|  | ***MD*** | 0.107 (0.006) | 0.111 (0.006) | 0.113 (0.007) | 0.149 (0.009) | 0.154 (0.008) | 0.158 (0.009) | 0.360 (0.022) | 0.372 (0.020) | 0.381 (0.022) | 0.107 (0.006) | 0.111 (0.006) | 0.113 (0.007) |
|  | *p-value* |  | 0.351 | ***0.031*** |  | 0.549 | ***0.033*** |  | 0.469 | ***0.028*** |  | 0.351 | ***0.031*** |
|  | ***aD*** | 0.118 (0.005) | 0.122 (0.006) | 0.124 (0.007) | 0.165 (0.008) | 0.170 (0.007) | 0.174 (0.009) | 0.396 (0.018) | 0.409 (0.018) | 0.418 (0.022) | 0.118 (0.005) | 0.122 (0.006) | 0.124 (0.007) |
|  | *p-value* |  | 0.273 | ***0.017*** |  | 0.283 | ***0.012*** |  | 0.271 | ***0.012*** |  | 0.273 | ***0.017*** |
|  | ***rD*** | 0.092 (0.007) | 0.096 (0.006) | 0.098 (0.007) | 0.130 (0.010) | 0.133 (0.008) | 0.137 (0.009) | 0.314 (0.024) | 0.324 (0.020) | 0.334 (0.022) | 0.092 (0.007) | 0.096 (0.006) | 0.098 (0.007) |
|  | *p-value* |  | 0.474 | 0.058 |  | 0.851 | 0.074 |  | 0.687 | 0.057 |  | 0.474 | 0.058 |
|  | ***Volume*** | 0.005 (0.000) | 0.005 (0.000) | 0.005 (0.000) | 0.021 (0.001) | 0.021 (0.001) | 0.021 (0.002) | 0.060 (0.003) | 0.060 (0.004) | 0.060 (0.005) | 0.005 (0.000) | 0.005 (0.000) | 0.005 (0.000) |
|  | *p-value* |  | 0.516* | 0.383* |  | 0.963* | 0.589* |  | 0.963* | 0.561* |  | 0.516* | 0.383* |

Columns report network metrics for each weight (rows). Values are expressed as mean (SD). Statistical analysis: *p*<0.05 was considered significant. Comparisons with controls are reported in *p-value* rows: *MCI* columns show *p-values* of the comparison between patients with mild cognitive impairment and controls, while *AD* columns show the ones between Alzheimer’s disease patients and controls. *Post hoc* pairwise comparisons were performed using Bonferroni or Mann-Whitney*. Note that all significant differences are between Alzheimer’s disease patients and controls.
