# Supplementary material for: Exploring Patterns of Alteration in Alzheimer's Disease Brain Networks: A Combined Structural and Functional Connectomics Analysis
Source: Front Neurosci. 2016 Sep 7;10:380. doi: 10.3389/fnins.2016.00380 (PMC5013043; doi:10.3389/fnins.2016.00380)
Supplement: Supplementary file 2 [file Presentation1.PPTX]

## Slide 1
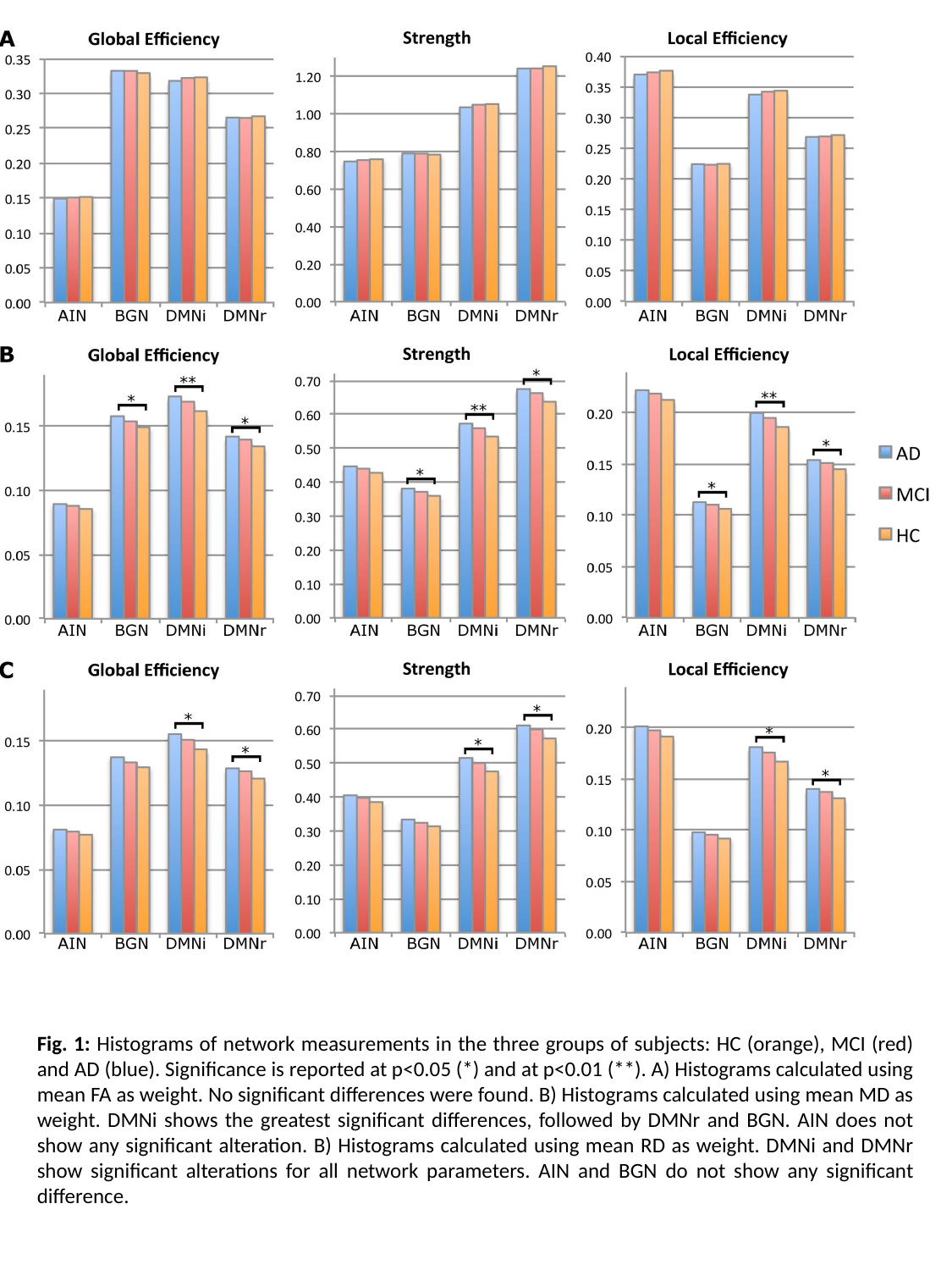

# Fig. 1: Histograms of network measurements in the three groups of subjects: HC (orange), MCI (red) and AD (blue). Significance is reported at p<0.05 (*) and at p<0.01 (**). A) Histograms calculated using mean FA as weight. No significant differences were found. B) Histograms calculated using mean MD as weight. DMNi shows the greatest significant differences, followed by DMNr and BGN. AIN does not show any significant alteration. B) Histograms calculated using mean RD as weight. DMNi and DMNr show significant alterations for all network parameters. AIN and BGN do not show any significant difference.
